# 4-(4-Chloro­benz­yl)-5-methyl-2-phenyl-1*H*-pyrazol-3(2*H*)-one

**DOI:** 10.1107/S1600536811013791

**Published:** 2011-04-16

**Authors:** Shaaban K. Mohamed, Mahmoud A. A. El-Remaily, Ahmed M. Soliman, Hossam Abdel-Ghany, Seik Weng Ng

**Affiliations:** a Division of Chemistry and Environmental Science, Manchester Metropolitan University, Manchester, England; b Department of Chemistry, Sohag University, Egypt; c Department of Chemistry, University of Malaya, 50603 Kuala Lumpur, Malaysia

## Abstract

The five-membered ring of the title compound, C_17_H_15_ClN_2_O, is almost planar (r.m.s. deviation = 0.008 Å), and its phenyl subsitutent is aligned at 34.9 (1)° with respect to this ring. The angle at the methyl­ene C atom is opened to 116.4 (2)°. In the crystal, adjacent mol­ecules are linked by an N—H⋯O hydrogen bond, generating a linear chain along the *a* axis.

## Related literature

For the synthesis, see: Pettinari *et al.* (1994 ▶).
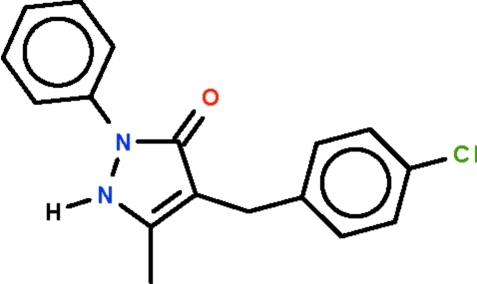


## Experimental

### 

#### Crystal data

C_17_H_15_ClN_2_O
*M* *_r_* = 298.76
Orthorhombic, 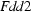

*a* = 23.1540 (3) Å
*b* = 43.8905 (6) Å
*c* = 5.6239 (1) Å
*V* = 5715.23 (15) Å^3^
*Z* = 16
Cu *K*α radiation
μ = 2.36 mm^−1^
*T* = 100 K
0.30 × 0.30 × 0.03 mm

#### Data collection

Agilent SuperNova Dual diffractometer with an Atlas detector
Absorption correction: multi-scan (*CrysAlis PRO*; Agilent, 2010 ▶) *T*_min_ = 0.538, *T*_max_ = 0.933
10182 measured reflections
2623 independent reflections
2611 reflections with *I* > 2σ(*I*)
*R*_int_ = 0.028

#### Refinement

*R*[*F* ^2^ > 2σ(*F* ^2^)] = 0.036
*wR*(*F* ^2^) = 0.097
*S* = 1.08
2623 reflections
195 parameters
1 restraint
H atoms treated by a mixture of independent and constrained refinement
Δρ_max_ = 0.21 e Å^−3^
Δρ_min_ = −0.41 e Å^−3^
Absolute structure: Flack (1983 ▶), 1011 Friedel pairs
Flack parameter: 0.000 (12)

### 

Data collection: *CrysAlis PRO* (Agilent, 2010 ▶); cell refinement: *CrysAlis PRO*; data reduction: *CrysAlis PRO*; program(s) used to solve structure: *SHELXS97* (Sheldrick, 2008 ▶); program(s) used to refine structure: *SHELXL97* (Sheldrick, 2008 ▶); molecular graphics: *X-SEED* (Barbour, 2001 ▶); software used to prepare material for publication: *publCIF* (Westrip, 2010 ▶).

## Supplementary Material

Crystal structure: contains datablocks global, I. DOI: 10.1107/S1600536811013791/bt5516sup1.cif

Structure factors: contains datablocks I. DOI: 10.1107/S1600536811013791/bt5516Isup2.hkl

Additional supplementary materials:  crystallographic information; 3D view; checkCIF report

## Figures and Tables

**Table 1:** Hydrogen-bond geometry (Å, °)

*D*—H⋯*A*	*D*—H	H⋯*A*	*D*⋯*A*	*D*—H⋯*A*
N2—H2⋯O1^i^	0.85 (3)	1.82 (3)	2.6516 (18)	165 (2)

Symmetry code: (i) 
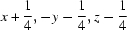
.

**Fig. 1.**
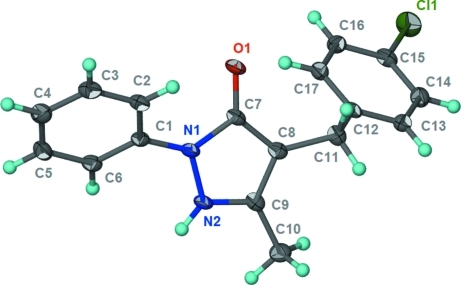
Anisotropic displacement ellipsoid plot (Barbour, 2001) of C17H15ClN2O at the 70% probability level; hydrogen atoms are drawn as spheres of arbitrary radius.

**Fig. 2.**
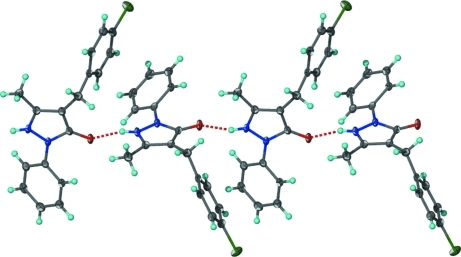
Hydrogen-bonded chain structure.

## supplementary crystallographic information

### Comment

3-Methyl-1-phenyl-4,5-dihydro-1*H*-5-pyrazolone possesses an active methylene linkage that undergoes condensation with aromatic aldehydes to yield compounds that react with metal salts (Pettinari *et al.*, 1994). In these organic compounds, the pyrazole ring is connected to the aromatic system (of the aldehyde precursor) by a methylene linkage. The five-membered ring of C_17_H_15_ClN_2_O (Scheme I) is planar, and its phenyl subsitutent is aligned at 34.9 (1) ° with respect to this ring. The angle at the methylene C atom is opened to 116.4 (2) ° (Fig. 1). Adjacent molecules are linked by an N–H···O hydrogen bond to generate a linear chain along the *a*-axis of the orthorhombic unit cell (Fig. 2).

### Experimental

3-Methyl-1-phenyl-4,5-dihydro-1*H*-5-pyrazolone (10 mmol)  and 4-chlorobenzaldehyde (10 mmol) along with few drops of concentrated hydrochloric acid were heated at 426 K in *N*,*N*-dimethylformamide (50 ml) for 8 h. The product was collected and recrystallized from ethanol; m.p. 471 K.

### Refinement

Carbon-bound H-atoms were placed in calculated positions [C—H 0.95 to 0.98 Å, *U*_iso_(H) 1.2 to 1.5*U*_eq_(C)] and were included in the refinement in the riding model approximation.

The amino H-atom was located in a difference Fourier map, and was freely refined.

### Crystal data

**Table:**

C_17_H_15_ClN_2_O	*F*(000) = 2496
*M**_r_* = 298.76	*D*_x_ = 1.389 Mg m^−^^3^
Orthorhombic, *F**d**d*2	Cu *K*α radiation, λ = 1.54184 Å
Hall symbol: F 2 -2d	Cell parameters from 8414 reflections
*a* = 23.1540 (3) Å	θ = 3.8–74.2°
*b* = 43.8905 (6) Å	µ = 2.36 mm^−^^1^
*c* = 5.6239 (1) Å	*T* = 100 K
*V* = 5715.23 (15) Å^3^	Plate, colorless
*Z* = 16	0.30 × 0.30 × 0.03 mm

### Data collection

**Table:**

Agilent SuperNova Dual diffractometer with an Atlas detector	2623 independent reflections
Radiation source: SuperNova (Cu) X-ray Source	2611 reflections with *I* > 2σ(*I*)
Mirror	*R*_int_ = 0.028
Detector resolution: 10.4041 pixels mm^-1^	θ_max_ = 74.3°, θ_min_ = 4.0°
ω scans	*h* = −24→28
Absorption correction: multi-scan (*CrysAlis PRO*; Agilent, 2010)	*k* = −53→54
*T*_min_ = 0.538, *T*_max_ = 0.933	*l* = −6→6
10182 measured reflections	

### Refinement

**Table:**

Refinement on *F*^2^	Secondary atom site location: difference Fourier map
Least-squares matrix: full	Hydrogen site location: inferred from neighbouring sites
*R*[*F*^2^ > 2σ(*F*^2^)] = 0.036	H atoms treated by a mixture of independent and constrained refinement
*wR*(*F*^2^) = 0.097	*w* = 1/[σ^2^(*F*_o_^2^) + (0.0771*P*)^2^ + 3.0966*P*] where *P* = (*F*_o_^2^ + 2*F*_c_^2^)/3
*S* = 1.08	(Δ/σ)_max_ = 0.001
2623 reflections	Δρ_max_ = 0.21 e Å^−^^3^
195 parameters	Δρ_min_ = −0.41 e Å^−^^3^
1 restraint	Absolute structure: Flack (1983), 1011 Friedel pairs
Primary atom site location: structure-invariant direct methods	Flack parameter: 0.000 (12)

### Fractional atomic coordinates and isotropic or equivalent isotropic displacement parameters (Å^2^)

**Table:**

	*x*	*y*	*z*	*U*_iso_*/*U*_eq_	
Cl1	0.122967 (18)	0.029555 (10)	0.59020 (10)	0.02795 (14)	
O1	0.20050 (5)	−0.13339 (3)	0.3426 (3)	0.0201 (3)	
N1	0.29664 (5)	−0.13658 (3)	0.2283 (3)	0.0152 (3)	
N2	0.34778 (6)	−0.12275 (3)	0.2955 (3)	0.0161 (3)	
H2	0.3806 (10)	−0.1243 (5)	0.228 (5)	0.019 (5)*	
C1	0.29551 (6)	−0.16087 (4)	0.0632 (3)	0.0143 (3)	
C2	0.25718 (7)	−0.18504 (4)	0.1001 (3)	0.0168 (3)	
H2A	0.2333	−0.1854	0.2373	0.020*	
C3	0.25436 (7)	−0.20838 (4)	−0.0648 (4)	0.0200 (4)	
H3	0.2279	−0.2247	−0.0416	0.024*	
C4	0.28990 (7)	−0.20825 (4)	−0.2645 (4)	0.0223 (4)	
H4	0.2875	−0.2243	−0.3776	0.027*	
C5	0.32888 (7)	−0.18450 (4)	−0.2971 (4)	0.0205 (4)	
H5	0.3537	−0.1845	−0.4316	0.025*	
C6	0.33170 (7)	−0.16074 (4)	−0.1343 (3)	0.0169 (3)	
H6	0.3582	−0.1445	−0.1577	0.020*	
C7	0.25237 (6)	−0.12512 (4)	0.3689 (3)	0.0150 (3)	
C8	0.27831 (7)	−0.10413 (4)	0.5272 (3)	0.0156 (3)	
C9	0.33640 (7)	−0.10305 (4)	0.4713 (3)	0.0162 (3)	
C10	0.38394 (7)	−0.08494 (4)	0.5800 (4)	0.0210 (4)	
H10A	0.4167	−0.0841	0.4700	0.031*	
H10B	0.3961	−0.0946	0.7290	0.031*	
H10C	0.3703	−0.0642	0.6126	0.031*	
C11	0.24961 (7)	−0.08915 (4)	0.7360 (3)	0.0178 (3)	
H11A	0.2789	−0.0863	0.8621	0.021*	
H11B	0.2200	−0.1033	0.7989	0.021*	
C12	0.22098 (6)	−0.05861 (4)	0.6893 (3)	0.0154 (3)	
C13	0.22392 (7)	−0.03570 (4)	0.8595 (3)	0.0172 (3)	
H13	0.2465	−0.0388	0.9985	0.021*	
C14	0.19446 (7)	−0.00837 (4)	0.8301 (4)	0.0193 (3)	
H14	0.1968	0.0072	0.9467	0.023*	
C15	0.16151 (7)	−0.00439 (4)	0.6266 (3)	0.0188 (4)	
C16	0.15824 (7)	−0.02657 (4)	0.4517 (3)	0.0184 (3)	
H16	0.1357	−0.0234	0.3128	0.022*	
C17	0.18871 (7)	−0.05357 (4)	0.4839 (3)	0.0175 (3)	
H17	0.1875	−0.0688	0.3640	0.021*	

### Atomic displacement parameters (Å^2^)

**Table:**

	*U*^11^	*U*^22^	*U*^33^	*U*^12^	*U*^13^	*U*^23^
Cl1	0.0289 (2)	0.0233 (2)	0.0317 (3)	0.01125 (15)	−0.00111 (18)	0.00169 (18)
O1	0.0092 (5)	0.0249 (6)	0.0261 (7)	−0.0009 (4)	0.0031 (5)	−0.0062 (6)
N1	0.0089 (6)	0.0176 (6)	0.0191 (7)	−0.0009 (5)	0.0009 (5)	−0.0015 (6)
N2	0.0086 (6)	0.0191 (6)	0.0206 (8)	−0.0025 (5)	0.0023 (6)	−0.0025 (6)
C1	0.0110 (6)	0.0154 (7)	0.0164 (8)	0.0031 (5)	−0.0018 (6)	0.0000 (6)
C2	0.0117 (6)	0.0168 (7)	0.0220 (9)	0.0010 (5)	0.0014 (7)	0.0001 (7)
C3	0.0148 (7)	0.0174 (8)	0.0277 (10)	0.0010 (6)	−0.0018 (7)	−0.0003 (7)
C4	0.0190 (8)	0.0216 (8)	0.0262 (10)	0.0053 (6)	−0.0021 (7)	−0.0062 (8)
C5	0.0167 (7)	0.0263 (8)	0.0185 (9)	0.0057 (6)	0.0021 (7)	−0.0009 (8)
C6	0.0128 (7)	0.0187 (7)	0.0191 (9)	0.0026 (5)	−0.0007 (6)	0.0021 (7)
C7	0.0118 (7)	0.0157 (7)	0.0176 (9)	0.0027 (5)	0.0021 (6)	0.0020 (6)
C8	0.0133 (7)	0.0159 (7)	0.0177 (9)	0.0015 (6)	0.0006 (6)	0.0020 (6)
C9	0.0147 (7)	0.0162 (7)	0.0177 (9)	0.0010 (6)	0.0024 (7)	0.0018 (7)
C10	0.0174 (7)	0.0213 (8)	0.0242 (10)	−0.0036 (6)	−0.0005 (7)	−0.0023 (8)
C11	0.0185 (7)	0.0174 (7)	0.0176 (9)	0.0018 (6)	0.0024 (7)	0.0005 (7)
C12	0.0104 (6)	0.0178 (8)	0.0178 (9)	−0.0013 (5)	0.0042 (6)	0.0001 (6)
C13	0.0145 (7)	0.0212 (7)	0.0157 (9)	−0.0005 (6)	0.0003 (6)	−0.0006 (7)
C14	0.0188 (7)	0.0190 (7)	0.0203 (9)	−0.0009 (6)	0.0023 (7)	−0.0030 (7)
C15	0.0150 (7)	0.0185 (8)	0.0229 (10)	0.0032 (6)	0.0029 (6)	0.0023 (7)
C16	0.0154 (7)	0.0250 (9)	0.0149 (8)	0.0001 (6)	−0.0009 (6)	0.0019 (7)
C17	0.0149 (7)	0.0200 (8)	0.0177 (9)	−0.0015 (6)	0.0028 (6)	−0.0030 (7)

### Geometric parameters (Å, °)

**Table:**

Cl1—C15	1.7488 (16)	C8—C9	1.382 (2)
O1—C7	1.263 (2)	C8—C11	1.501 (2)
N1—N2	1.3833 (18)	C9—C10	1.489 (2)
N1—C7	1.389 (2)	C10—H10A	0.9800
N1—C1	1.414 (2)	C10—H10B	0.9800
N2—C9	1.340 (2)	C10—H10C	0.9800
N2—H2	0.85 (3)	C11—C12	1.519 (2)
C1—C6	1.391 (2)	C11—H11A	0.9900
C1—C2	1.398 (2)	C11—H11B	0.9900
C2—C3	1.383 (3)	C12—C13	1.390 (2)
C2—H2A	0.9500	C12—C17	1.394 (3)
C3—C4	1.392 (3)	C13—C14	1.390 (2)
C3—H3	0.9500	C13—H13	0.9500
C4—C5	1.391 (3)	C14—C15	1.386 (3)
C4—H4	0.9500	C14—H14	0.9500
C5—C6	1.389 (3)	C15—C16	1.386 (3)
C5—H5	0.9500	C16—C17	1.391 (2)
C6—H6	0.9500	C16—H16	0.9500
C7—C8	1.415 (2)	C17—H17	0.9500
			
N2—N1—C7	108.49 (14)	C8—C9—C10	130.13 (17)
N2—N1—C1	121.74 (13)	C9—C10—H10A	109.5
C7—N1—C1	129.29 (13)	C9—C10—H10B	109.5
C9—N2—N1	108.46 (13)	H10A—C10—H10B	109.5
C9—N2—H2	123.9 (16)	C9—C10—H10C	109.5
N1—N2—H2	127.2 (16)	H10A—C10—H10C	109.5
C6—C1—C2	120.26 (16)	H10B—C10—H10C	109.5
C6—C1—N1	120.65 (14)	C8—C11—C12	116.41 (15)
C2—C1—N1	119.09 (15)	C8—C11—H11A	108.2
C3—C2—C1	119.48 (16)	C12—C11—H11A	108.2
C3—C2—H2A	120.3	C8—C11—H11B	108.2
C1—C2—H2A	120.3	C12—C11—H11B	108.2
C2—C3—C4	120.66 (16)	H11A—C11—H11B	107.3
C2—C3—H3	119.7	C13—C12—C17	118.84 (15)
C4—C3—H3	119.7	C13—C12—C11	119.89 (15)
C5—C4—C3	119.54 (17)	C17—C12—C11	121.16 (16)
C5—C4—H4	120.2	C12—C13—C14	121.22 (16)
C3—C4—H4	120.2	C12—C13—H13	119.4
C6—C5—C4	120.39 (17)	C14—C13—H13	119.4
C6—C5—H5	119.8	C15—C14—C13	118.51 (16)
C4—C5—H5	119.8	C15—C14—H14	120.7
C5—C6—C1	119.65 (15)	C13—C14—H14	120.7
C5—C6—H6	120.2	C14—C15—C16	121.82 (15)
C1—C6—H6	120.2	C14—C15—Cl1	119.01 (13)
O1—C7—N1	122.06 (16)	C16—C15—Cl1	119.16 (14)
O1—C7—C8	131.63 (15)	C15—C16—C17	118.58 (17)
N1—C7—C8	106.30 (13)	C15—C16—H16	120.7
C9—C8—C7	107.01 (15)	C17—C16—H16	120.7
C9—C8—C11	126.41 (16)	C16—C17—C12	121.00 (17)
C7—C8—C11	126.12 (15)	C16—C17—H17	119.5
N2—C9—C8	109.69 (14)	C12—C17—H17	119.5
N2—C9—C10	120.14 (14)		
			
C7—N1—N2—C9	−0.45 (19)	N1—C7—C8—C11	−170.82 (16)
C1—N1—N2—C9	−173.26 (14)	N1—N2—C9—C8	1.6 (2)
N2—N1—C1—C6	−39.1 (2)	N1—N2—C9—C10	179.61 (16)
C7—N1—C1—C6	149.68 (17)	C7—C8—C9—N2	−2.2 (2)
N2—N1—C1—C2	141.26 (16)	C11—C8—C9—N2	170.45 (15)
C7—N1—C1—C2	−29.9 (2)	C7—C8—C9—C10	−179.87 (18)
C6—C1—C2—C3	−1.8 (2)	C11—C8—C9—C10	−7.3 (3)
N1—C1—C2—C3	177.77 (15)	C9—C8—C11—C12	97.3 (2)
C1—C2—C3—C4	1.0 (2)	C7—C8—C11—C12	−91.5 (2)
C2—C3—C4—C5	0.5 (3)	C8—C11—C12—C13	−142.06 (16)
C3—C4—C5—C6	−1.2 (3)	C8—C11—C12—C17	41.7 (2)
C4—C5—C6—C1	0.4 (2)	C17—C12—C13—C14	1.3 (2)
C2—C1—C6—C5	1.1 (2)	C11—C12—C13—C14	−174.99 (15)
N1—C1—C6—C5	−178.48 (15)	C12—C13—C14—C15	0.4 (2)
N2—N1—C7—O1	178.60 (15)	C13—C14—C15—C16	−1.3 (2)
C1—N1—C7—O1	−9.3 (3)	C13—C14—C15—Cl1	178.64 (13)
N2—N1—C7—C8	−0.87 (19)	C14—C15—C16—C17	0.5 (3)
C1—N1—C7—C8	171.23 (16)	Cl1—C15—C16—C17	−179.46 (13)
O1—C7—C8—C9	−177.57 (18)	C15—C16—C17—C12	1.3 (2)
N1—C7—C8—C9	1.82 (19)	C13—C12—C17—C16	−2.2 (2)
O1—C7—C8—C11	9.8 (3)	C11—C12—C17—C16	174.10 (15)

### Hydrogen-bond geometry (Å, °)

**Table:**

*D*—H···*A*	*D*—H	H···*A*	*D*···*A*	*D*—H···*A*
N2—H2···O1^i^	0.85 (3)	1.82 (3)	2.6516 (18)	165 (2)

Symmetry codes: (i) *x*+1/4, −*y*−1/4, *z*−1/4.
